# Effects of Vertical Transmission of Respiratory Viruses to the Offspring

**DOI:** 10.3389/fimmu.2022.853009

**Published:** 2022-03-14

**Authors:** Sara Manti, Salvatore Leonardi, Fariba Rezaee, Terri J. Harford, Miriam K. Perez, Giovanni Piedimonte

**Affiliations:** ^1^ Pediatric Pulmonology Unit, Department of Clinical and Experimental Medicine, University of Catania, Catania, Italy; ^2^ Inflammation and Immunity, Lerner Research Institute, Cleveland Clinic Foundation, Cleveland, OH, United States; ^3^ Center for Pediatric Pulmonology, Cleveland Clinic Children’s, Cleveland, OH, United States; ^4^ Department of General Pediatrics, Cleveland Clinic Children’s, Cleveland, OH, United States; ^5^ Department of Pediatrics, Biochemistry and Molecular Biology, Tulane University, New Orleans, LA, United States

**Keywords:** influenza virus, respiratory syncytial virus – RSV, severe acute respiratory syndrome coronavirus-2 (SARS-CoV2), vertical transmission, intrauterine exposure

## Abstract

Overt and subclinical maternal infections in pregnancy can have multiple and significant pathological consequences for the developing fetus, leading to acute perinatal complications and/or chronic disease throughout postnatal life. In this context, the current concept of pregnancy as a state of systemic immunosuppression seems oversimplified and outdated. Undoubtedly, in pregnancy the maternal immune system undergoes complex changes to establish and maintain tolerance to the fetus while still protecting from pathogens. In addition to downregulated maternal immunity, hormonal changes, and mechanical adaptation (e.g., restricted lung expansion) make the pregnant woman more susceptible to respiratory pathogens, such as influenza virus, respiratory syncytial virus (RSV), and severe acute respiratory syndrome coronavirus-2 (SARS-CoV-2). Depending on the infectious agent and timing of the infection during gestation, fetal pathology can range from mild to severe, and even fatal. Influenza is associated with a higher risk of morbidity and mortality in pregnant women than in the general population, and, especially during the third trimester of pregnancy, mothers are at increased risk of hospitalization for acute cardiopulmonary illness, while their babies show higher risk of complications such as prematurity, respiratory and neurological illness, congenital anomalies, and admission to neonatal intensive care. RSV exposure *in utero* is associated with selective immune deficit, remodeling of cholinergic innervation in the developing respiratory tract, and abnormal airway smooth muscle contractility, which may predispose to postnatal airway inflammation and hyperreactivity, as well as development of chronic airway dysfunction in childhood. Although there is still limited evidence supporting the occurrence of vertical transmission of SARS-CoV-2, the high prevalence of prematurity among pregnant women infected by SARS-CoV-2 suggests this virus may alter immune responses at the maternal-fetal interface, affecting both the mother and her fetus. This review aims at summarizing the current evidence about the short- and long-term consequences of intrauterine exposure to influenza, RSV, and SARS-CoV-2 in terms of neonatal and pediatric outcomes.

## Introduction

**Figure 1:**
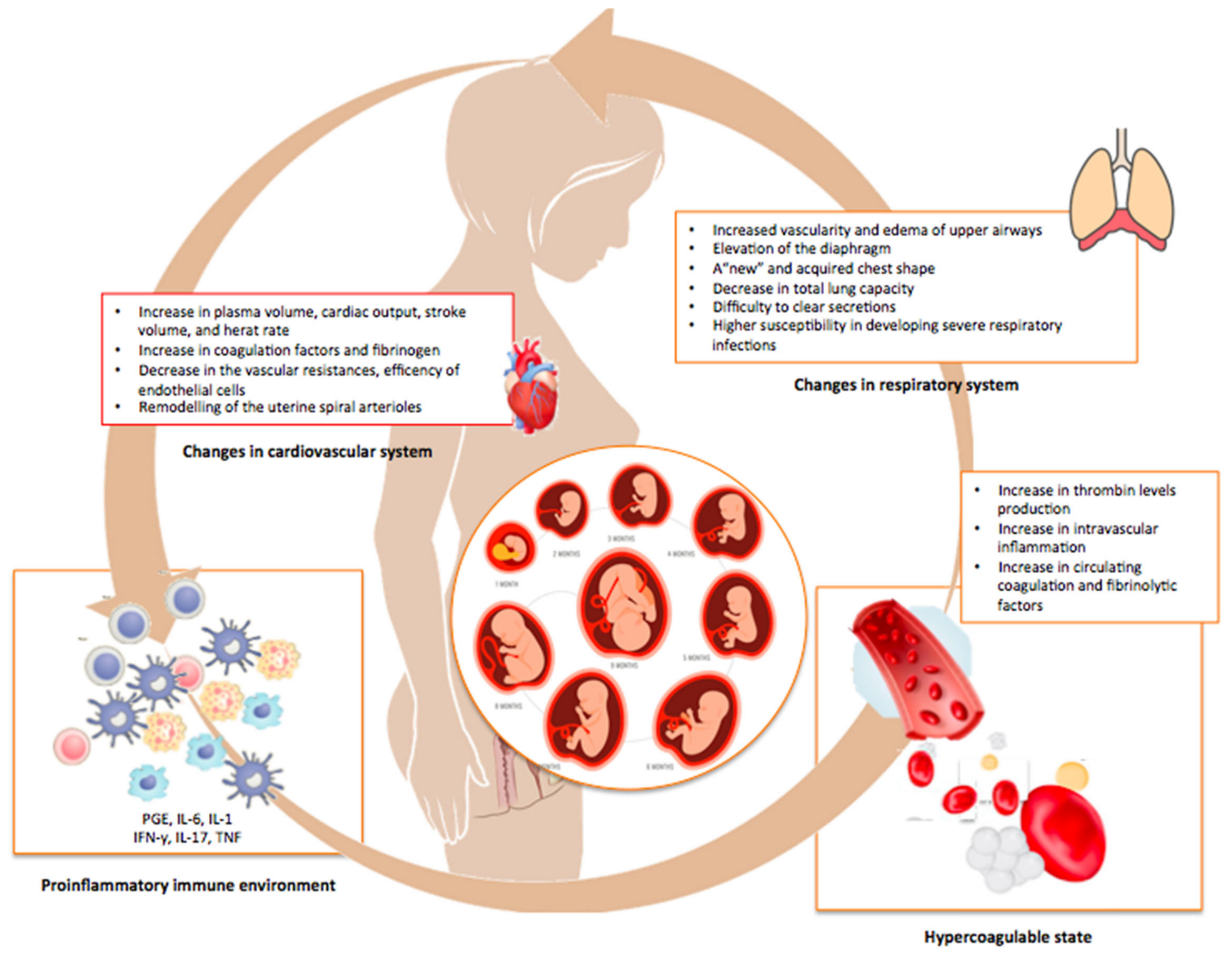
Physiologic adjustments occurring during pregnancy. This illustration summarizes the multiple and profound changes in the immune, pulmonary, cardiovascular, and coagulation systems of the mother occurring during normal pregnancy in order to avoid rejection and promote the growth of the fetus.

In pregnancy, several profound physiological changes occur that involve in particular the immune, respiratory, cardiovascular, and hormonal systems of the mother (**Figure 1**). Among other consequences, all these events make pregnant women more susceptible to respiratory pathogens, such as influenza, respiratory syncytial virus (RSV), and severe acute respiratory syndrome coronavirus-2 (SARS-CoV-2) (1, 2). Depending on the viral agent, the timing of infection during gestation, and the efficiency of viral neutralization and clearance by the host immune system, the infection can follow different trajectories and the consequences for the mother and the fetus can range from mild to severe, and even fatal (1, 2).

The fetus and placenta necessarily express paternal antigens that are disparate (“non-self”) from the mother. Yet the mother’s immune system does not reject a developing fetus as foreign tissue. To avoid rejection and permit fetal growth, the maternal immune system undergoes profound modifications resulting in impaired responses to infections, including: a shift in the CD4+ T cell population toward the Th2 phenotype, which compromises the clearance of infected cells; a reduction in natural killer (NK) cells that play a critical role in viral clearance; a decrease in serum plasmacytoid dendritic cells (pDCs), critical for the synthesis of interferon type 1 (IFN-1); changes in pregnancy-related hormones, such as progesterone and adiponectin, which can influence the immune response to viral infections; and functional loss of pattern recognition Toll-like receptors (TLRs), a family of innate immune receptors also recognizing viral pathogens (1, 2).

The immuno-inflammatory events occurring at the maternal-fetal interface are critical for the positive outcome of a pregnancy. Such events are organized in three distinct stages: a proinflammatory stage (implantation and placentation, occurring in the first trimester); an anti-inflammatory stage (fetal growth, occurring in the second trimester); and a second proinflammatory stage (initiation of parturition, occurring in the third trimester) (3).

Between days 19 and 23 of the menstrual cycle, the uterus becomes receptive to the implant of the blastocyst, which invades the endometrial tissue. This process forms “an open wound” requiring a robust inflammatory environment that involves cellular infiltration and repair, as well as several adhesion molecules, growth factors, chemokines, and cytokines (including interleukin (IL)-6, IL-8, and tumor necrosis factor (TNF)α) (4). The latter can be secreted by endometrial and immune cells recruited at the implantation site, such as uterine-specific natural killer (NK) cells, macrophages, and dendritic cells (DCs).

NK cells are innate immune effectors exerting cytolytic activity against infected cells without human leukocyte antigen (HLA) restriction. At the time of implantation, decidual NK (dNK) cells are predominant in the endometrium and represent up to 70% of the local immune cells (5). While the rationale for this event is not yet understood, researchers have hypothesized two mechanisms to explain the pooling of dNK cells during pregnancy: first, the proliferation of local tissue-resident NK cells at the time of decidualization, and secondly, the recruitment of peripheral NK cells, required for vascular remodeling of the placenta (5). Recently, three different subsets of dNK cells have been identified (dNK1, dNK2, dNK3), which are involved in trophoblast invasion and immunomodulatory pathways involving T cells, stromal cells, and myeloid cells (6). NK cells are also prime candidates for counteracting placental infection. Previous studies have shown that dNK cells express high levels of the antimicrobial peptide granulysin and selectively transfer this peptide to the extra-villous trophoblast *via* nanotubes for the selective killing of infectious agents (7). Moreover, dNK cells induce the release of proinflammatory cytokines/chemokines and angiogenic factors (8, 9). Notably, dNK cells remain abundant until the end of the second trimester, and their number gradually decreases afterward (8, 9).

Several studies have also revealed the critical role of circulating and decidual DCs during pregnancy. Circulating DCs are classified into myeloid and plasmacytoid (mDCs and pDCs). While mDCs are primarily involved in IL-10 production and Treg polarization from Th1 to Th2, pDCs participate in the innate antiviral response by releasing type I IFN. Although decidual DCs account for less than 2% of decidual leukocytes, they play a critical role throughout pregnancy by interacting with multiple immune effectors. Because of the common origin, DCs and macrophages share similar phenotypical and functional features (10). In the first trimester of pregnancy, high expression of IL-1β and TNF-α promotes the recruitment of macrophages which, in turn, trigger the recruitment of DCs into the decidua by releasing macrophage growth factor colony-stimulating factor (M-CSF). Subsequently, macrophages can differentiate into decidual DCs with immunosuppressive properties during the second trimester or in cells with immunostimulatory properties during the last trimester of pregnancy. These findings are consistent with the potential of macrophages to evolve into proinflammatory (M1) or anti-inflammatory (M2) phenotypes, with the M1 phenotype predominating in the first trimester of pregnancy (implantation and trophoblast invasion) and the M2 phenotype predominating both in the second and third trimesters. Lastly, another shift from the M2 to the M1 phenotype occurs again close to the parturition (11).

By inducing transforming growth factor β (TGF-β)-mediated proliferation and differentiation of T cells into Treg cells, decidual DCs promote tolerogenic responses, Th2-type cytokines-mediated immunosuppressive functions, and reduce cytotoxic NK cells, thereby favoring trophoblast invasiveness and preventing fetal rejection (10). In addition, decidual DCs display a close cross-talk with NK cells both directly and indirectly. For example, previous studies have found cell-to-cell communication between decidual DCs and NKs, as more than 60% of decidual DCs are found in close proximity with NKs in the human decidua (12). In particular, through modulation of IL-15 and IL-12 mRNA and protein expression, decidual DCs decrease the number of NKs and the release of IFN-y (10).

Based on their cell surface receptors, interferons (IFNs) are classified into type-I (IFN-α, β, δ, ϵ, τ), type-II (IFN-γ), and type-III (IFN-λ1, λ2, λ3). Type-I and II IFNs are synthesized by all cell types, whereas type-III is released only by epithelial and dendritic cells (13). All of them are involved in the normal fetal development and progression of pregnancy and antiviral immune responses (14). Following the release from virus-infected cells, INFs bind their specific receptors and trigger receptor endocytosis. This interaction leads to activation first of intracellular mediators, such as receptor-associated tyrosine kinases Janus kinase I (JAK-1) and tyrosine kinase 2 (TYK2), and then of transcription factors including signal transducer and activator of transcription (STAT)1 and STAT2, which are associated with IFN regulatory factor 9 (IRF9). After translocating to the nucleus, IRF9 binds IFN-stimulated response elements (IRSEs) and modulates the transcription of IFN-stimulated genes (ISGs) that coordinate the response to infection (14). In addition to inhibiting the transmission of infections from the mother to the fetus, IFNs signaling plays a critical role throughout pregnancy in promoting a physiologic gestation, initiating uterine vascular modifications, and ensuring decidual integrity (15).

However, when its action is excessive or sustained, type-I IFN can cause deleterious effects during pregnancy. Thus, physiologic regulation of its expression and activity is critical for an effective immune response and maintenance of homeostasis. The regulation of type-I IFN pathways includes: 1. modulation of receptors involved in signaling (TYRO3, AXL, and MER); 2. factors released from commensal bacteria *via* interaction with TLR4 (16); and 3. mechanisms suppressing type-I IFN-mediated apoptosis. In this context, several studies have reported that loss in type-I IFN (IFN-β) in the placenta promotes viral replication and hypersensitivity to bacterial products, resulting in fetal infection and maternal mortality (17). Unlike IFN-α and β, IFN-ϵ is released constitutively in the female reproductive tract of humans, although fluctuation in its levels has been reported. Currently, the role of IFN-ϵ during pregnancy is not well understood, but it may also protect the fetus from ascending infections (18). Type-I IFN-τ, released by the trophoblast, acts as a recognition factor for maternal hormone levels and induces ISGs expression in the maternal endometrium (19). Similar to IFN-τ, IFN-δ has been shown to induce maternal ISGs expression before trophoblast attachment in swine models (20).

**Figure 2:**
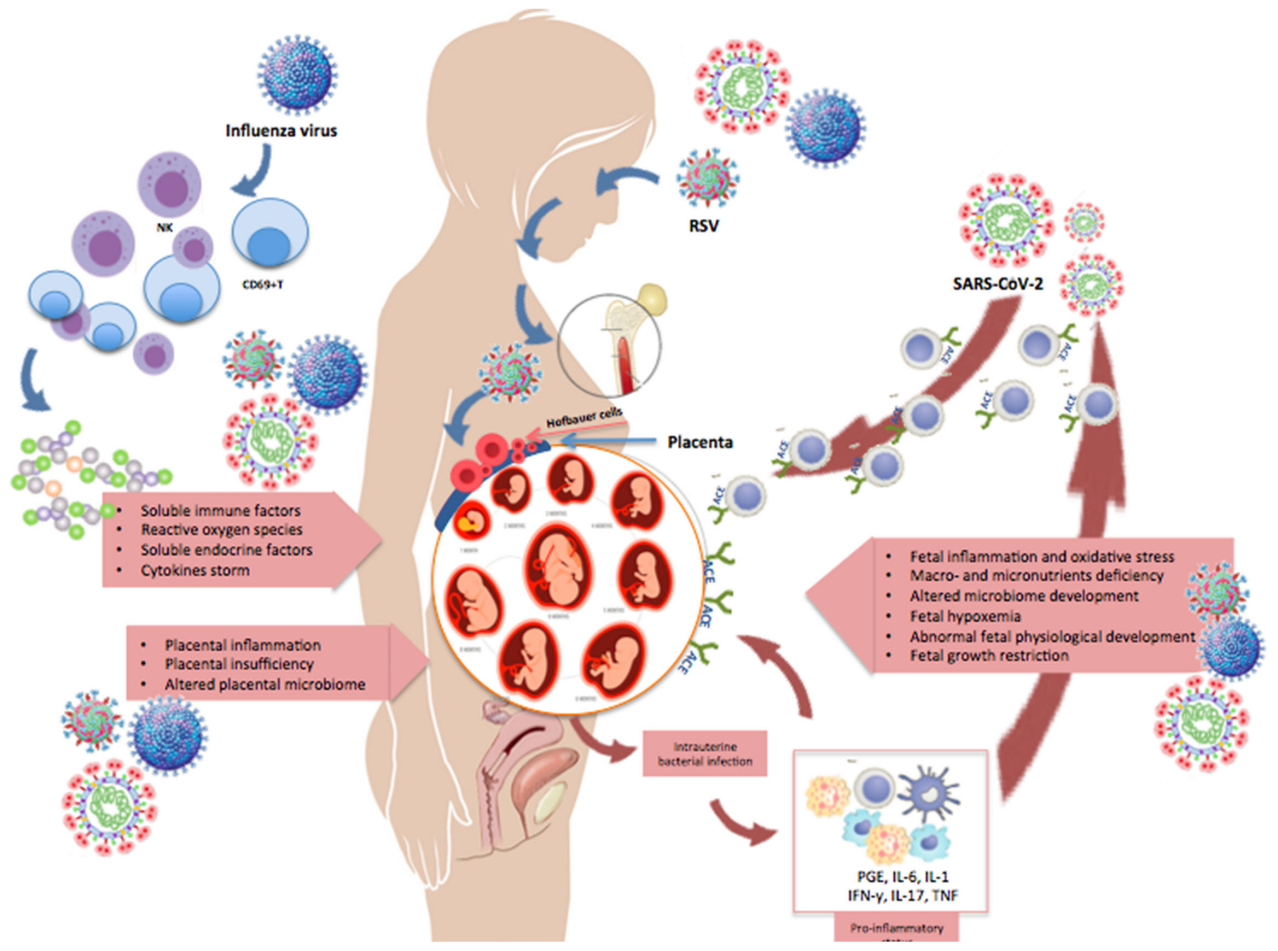
Changes occurring in pregnancy during maternal infection. This illustration shows some of the changes occurring in pregnancy during a maternal infection with influenza, RSV, or SARS-CoV-2. Pregnant women may develop an explosive inflammatory response to the virus (cytokine storm), which is characterized by significant increase in NK- and T-cells, CD69+ lymphocytes, and expression of pro- and anti-inflammatory cytokines and chemokines. Moreover, the virus can spread from the maternal respiratory tract *via* the bloodstream and reach the placenta and amniotic fluid, causing chorioamnionitis with degeneration of the vascular endothelium, placental trophoblasts, decidual and amniotic cells. The figure shows that viral antigens and genomic sequences can disseminate to bone marrow stromal cells and other extrapulmonary tissues, like the placenta. Receptors necessary for viral entry (e.g., ACE2) have also been detected on placental cells, supporting the possibility of maternal-fetal transmission of respiratory viruses.

The immunological changes occurring during pregnancy further impair a lung function already affected by multiple physiological adaptations, such as the diaphragm elevation and the modified shape of the maternal chest that cause a decrease in total lung capacity and compromise the clearance of secretions (21). Also, cardiovascular adaptations like increased blood volume and cardiac output, decreased vascular resistance, and remodeling of the uterine spiral arterioles in placental villi are all critical determinants for pregnancy outcomes (22). By impairing physical defenses, viruses can break the protective syncytiotrophoblast layer and spread directly to the extra-villous trophoblast or, *via* paracellular or transcellular transport, reach the fetal circulation (22). The increased thrombin production and intravascular inflammation, with a parallel increase in circulating coagulation and fibrinolytic factors, can further contribute to the virulence of the infection (23, 24). Overall, the combination of structural, physiologic, and immunologic changes occurring in pregnancy may exert additive or synergistic effects on the clinical evolution of a maternal viral infections and, consequently, on the fetus (**Figure 2**).

It must be emphasized that the detection of viral pathogens in the placenta does not necessarily imply an active placental infection and, even when the infection of placental cells is confirmed, the vertical transmission of viruses is not a necessary consequence. Furthermore, even when a direct fetal infection occurs, fetal responses to viral pathogens are highly heterogeneous and not always associated with persistent or permanent injury. Finally, the ultimate susceptibility to infections critically depends on the virulence of the invading vira pathogen and its interactions with multiple host defense mechanisms.

On the other hand, a maternal infection - especially within critical developmental windows - may damage the fetus even without direct invasion of fetal tissues, *via* activation of maternal inflammation and immunity with synthesis of soluble mediators and mobilization of cellular effectors able to cross the placental filter, or by simply impairing placental function and limiting the passage of nutrients and other factors essential for fetal growth. This review aims at summarizing the current knowledge about the effects of intrauterine exposure to influenza, RSV, and SARS-CoV-2 on neonatal and pediatric outcomes.

## Influenza Virus

Influenza virus is a single-stranded RNA virus belonging to the *Orthomyxoviridae* family. Except for influenza type C, which is antigenically stable, types A and B are responsible for annual epidemics in humans due to antigenic drift of the surface glycoproteins hemagglutinin (HA) and neuraminidase (NA), as well as the occurrence of antigenic shift (25). Epidemics of influenza are globally distinguished into seasonal and pandemic types. While seasonal influenza peaks in the fall and winter, pandemic influenza is caused by virus strains antigenically different from seasonal strains, such as the Spanish (H1N1) in 1918 (26), Asian (H2N2) in 1967 (27), Hong Kong (H3N2) in 1967 (28), H5N1 in 1997 (29), H1N1 in 2009 (30), and H7N9 in 2013 (31).

Globally, epidemic influenza is responsible for approximately 300,000 to 650,000 deaths every year, with hospitalizations and deaths primarily due to respiratory complications like pneumonia, exacerbation of underlying cardiac or pulmonary conditions, rhabdomyolysis, and acute renal failure (32). All these complications result from direct and indirect effects of the virus on the respiratory system and the ensuing host immune response (25). Through HA, the virus binds sialic acid residues expressed on the surface of respiratory epithelial cells, enters the cells, and starts replication of its genomic RNA that guides the synthesis of new viral ribonucleoprotein complexes. These ribonucleoproteins are then encapsidated into complete virions that egress and spread to adjacent cells, helped by the viral NA reducing the viscosity of the mucous film coating the respiratory mucosa (25).

In parallel, the virus can modulate host immune responses by inducing the release of anti-inflammatory cytokines, such as interleukin (IL)-10, which in turn impair immune defenses and increase host susceptibility to bacterial superinfections (33). The innate immune response - primarily driven by neutrophils, macrophages, and pDCs - provides a first barrier against the infection. Subsequently, pattern-recognition receptors (PRRs) detecting circulating viral components, as well as CD4+ and CD8+ T-cells are activated to start the adaptive response. However, in pregnancy, while humoral immunity is somewhat preserved, cell-mediated immunity is significantly impaired, which results in increased susceptibility of pregnant women to influenza infection (34).

Consistently, during the Spanish flu of 1918 higher mortality rates were observed in pregnant women (35), and similar epidemiologic data collected during the H1N1 influenza of 2009 showed that pregnant women were at higher risk of developing a severe infection when compared to the general population, resulting in increased morbidity, hospital admissions, and mortality in all three trimesters pregnancy. Notably, multiparity, multiple pregnancy, asthma, obesity, black or other minority group ethnicity, and smoking were reported as significant risk factors for hospital admission (36). Although pregnant women were less than 1% of the global population during the H1N1 influenza pandemic, they accounted for 5% of all deaths (37), ranging from 7.1% in the first trimester, to 26.8% in the second, and a peak of 64.3% in the third trimester (30, 37).

Nevertheless, it has also been reported that pregnant women can develop a remarkable pro-inflammatory response to influenza virus, which is characterized by a significant increase in peripheral NK and T cells, CD69+ lymphocytes, as well as pro- and anti-inflammatory cytokines and chemokines. Accordingly, *in vivo* animal studies have shown that influenza infection during pregnancy results both in a “vascular storm” (38) and in a “cytokine storm” (39) involving proinflammatory mediators, Ly6C monocytes, neutrophils, and T cells. These inflammatory events impair blood supply and lead to placental and fetal brain hypoxia, resulting in higher morbidity and mortality rates for pregnant dams, as well as in perinatal complications for the offspring (38, 39). Probably, also the deficiency in total immunoglobulin (Ig)G and IgG2 levels occurring during pregnancy contributes to influenza-mediated complications (40). Hence, several studies have shown that influenza can injury the feto-placental unit directly and indirectly. The virus spreads through the maternal bloodstream and reaches the placenta and amniotic fluid, causing chorioamnionitis with degeneration of vascular endothelial cells, placental trophoblasts, decidual and amniotic cells. Moreover, the virus exerts a direct cytopathic effect leading to the apoptosis of chorion cells (41, 42).

Based on the data discussed above, it is not surprising that several studies have reported increased risk of adverse pregnancy outcomes associated with influenza infection, but such findings were not confirmed by other studies (43). More specifically, it has been reported an increased occurrence of spontaneous miscarriage, preterm delivery, cesarean delivery, pneumonia, maternal hyperthermia, and post-partum hospital length of stay (43–46). Similarly, the effects of maternal influenza infection on the fetus are also under investigation (43–46), but evidence of the effects of inter-pandemic influenza on fetal outcomes has varied among different countries and the published studies have shown substantial biases (43). In general, when comparing non-infected to influenza-infected pregnant women, the general consensus is that the latter group has a higher risk for prematurity, small for gestational age (SGA) birth, low birth weight (LBW), stillbirth, hydrocephaly, cleft lip, neural tube and congenital heart defects in (43, 47–51).

Notably, by analyzing United States (US)-wide data, Dorélien et al. (47) reported that while the risk for neonatal and infant mortality, as well as preterm birth, was higher with first trimester infections, it decreased during the second trimester. However, a significant increase in LBW was found in the first trimester. A significant increase in LBW and abnormal fetal length was also found with third trimester infections, which were also associated with increased prematurity risk. This association of adverse outcomes with the timing of infection can be explained with the “Barker hypothesis”, which postulates that *not-programmed* events during fetal development can have long-lasting health effects in accordance with to the organ systems developing at the specific time of maternal infection (52). As expected, prematurity and LBW resulted in other perinatal complications such as jaundice, pneumonia, neurodevelopmental abnormalities, and neonatal and infant mortality.

Interestingly, this population was also at increased risk for developing chronic diseases both in early life and adult life (43, 51, 53). A multivariate analysis evaluating the long-term outcomes of offspring born from women exposed to influenza during pregnancy and followed up to 80  months after birth found that, while *in utero* exposure to the virus was associated with LBW, a higher proportion of the same population suffered from overweight and insulin-resistance. Moreover, hypertension, kidney disease, type 2 diabetes, cardiovascular disease, and other metabolic diseases occurred more frequently in the cohort of children born to influenza-infected mothers when compared to age-matched controls born to not-infected mothers (43, 53–55).

## Respiratory Syncytial Virus (RSV)

RSV is a RNA Pneumovirus belonging to the *Paramyxoviridae* family. Its genome is enclosed in a capsid of eleven proteins and protected by a lipid envelope. The spikes protruding outside the envelope are made of the glycoproteins G and F (fusion) that bind to airway epithelial cells and promote fusion of the viral envelope to the of host cell membranes generating the characteristic syncytia (56). RSV A and B are the two strains detected during epidemic seasons, and the most frequent viral agents of bronchiolitis and pneumonia in infants and preschool children, responsible for 24 hospitalizations per 1,000 infants and 1 million deaths worldwide every year.

RSV is horizontally transmitted through direct contact of the nasopharyngeal or conjunctival mucosa with respiratory secretions of infected patients. Following replication in the nasal mucosa, RSV spreads throughout the respiratory tract causing upper respiratory symptoms like clear rhinorrhea and sneezing, as well as lower respiratory tract symptoms like cough, airflow obstruction, wheeze, and increased use of accessory respiratory muscles resulting from inflammation, edema, and necrosis of the respiratory mucosa (56). Yearly reinfections are frequent because the immune memory for this virus is short lived, but are usually limited to cold-like illnesses milder than the first infection.

RSV infection in pregnant women, may be vertically transmitted to the offspring and lead to adverse perinatal outcomes. Limited epidemiological studies suggest that RSV infection is generally uncommon in pregnant women, occurring in 2% to 9% of pregnancies (57, 58). Frequent clinical manifestations include fever, with a median duration of 2-3 days, and upper airways symptoms like rhinorrhea and sore throat with a median duration of 4 days. Less frequently, wheezing, shortness of breath, hypoxemia, and other symptoms of lower respiratory tract involvement are noted, occasionally requiring hospitalization (58, 59). Indeed, a prospective study reported that 50% of RSV-positive pregnant women developed a severe infection requiring more than 3 days of hospitalization, and 40% of them was diagnosed as having pneumonia. Interestingly, preterm birth occurred more frequently in RSV-positive pregnant women when compared to RSV-negative controls. Also, complications were more frequent with third trimester infections (59). In a more recent study, authors showed that 10 out of 20 pregnant women with RSV infection were hospitalized for pneumonia, atelectasis, respiratory failure, or sepsis (60), and 9 of them had at least one comorbid condition such as asthma, obesity, or co-infection. Again, 9 out of 10 pregnant women were hospitalized during the third trimester of pregnancy, a period in which major physiologic changes occur in the maternal respiratory and immune systems. It should be noted that the true incidence of RSV infection in pregnancy might be underdiagnosed due to infrequent testing.

Multiple reports of RSV antigens and genomic sequences in extrapulmonary tissues (e.g., human bone marrow stromal cells) of infected human subjects support the hypothesis that this virus has access to the systemic circulation, which implies the possible transmission of this infection from the mother’s respiratory tract to the fetus in pregnancy (61, 62). In an experimental rodent model of maternal infection at mid-gestation, RSV genome was found in 30% of fetuses, as well as in the lungs of 40% of newborns and 25% of adults exposed *in utero* (63). Both exposed (i.e., born from RSV-infected mother but without RSV RNA detected in lungs after birth) and infected (i.e., with RSV RNA detected in lungs) newborn pups showed evidence of impaired Th1 immunity, aberrant cholinergic innervation, and airway hyperreactivity to both methacholine and nerve stimulation following postnatal RSV reinfection (63–65). These experimental data were successively supported by an independent human study that showed droplet digital PCR (ddPCR) evidence of RSV genome in cord blood mononuclear cells from 26 of 45 (57.7%) term infants delivered by healthy mothers recruited antenatally (66). As cord blood is of exclusive fetal origin, any virus isolated from cord blood can only be transmitted by the mother during the pregnancy, and this test is universally accepted as the “gold standard” of vertical transmission. Indeed, consistent with the epidemiologic profile of RSV epidemics, samples testing positive for RSV were identified across all birth seasons, but a greater number of RSV positive samples was observed in winter compared to non-winter birth months.

Later, RSV genome was detected in human cord blood mononuclear cells and from the peripheral blood of a newborn presenting with severe respiratory distress immediately after delivery from a mother with serological and clinical evidence of RSV infection during the third trimester of pregnancy (67). Furthermore, Bokum et al. (68) showed in RSV-infected human placentas that Hofbauer cells (fetal migratory M2-type macrophages located in the chorionic villous stroma) support RSV infection for up to 30 days and are also able to transinfect naïve epithelial cell through a contact-dependent mechanism. Because Hofbauer cells are mobile and localized in the proximity of the fetal vasculature, they can cross into the fetal circulation and spread hematogenously to the fetal lung, as shown with other vertically transmitted viruses like Zika (68).

Transplacental infection results in complications of pregnancy and adverse birth outcomes. The first case of vertical RSV transmission was described in a 35 weeks infant delivered *via* cesarean delivery due to reduced fetal movement. Following delivery, the baby experienced respiratory distress syndrome and tested positive for RSV, as diagnosed by high-titer serum anti-RSV IgM and IgA as well as by PCR amplification of RSV RNA. In parallel, RSV infection was also detected in the mother and confirmed by positive anti-RSV IgM, IgA, and IgG. The infant required ventilatory support and, at 17 days of life, tested negative for RSV (67).

In a similar study, Chu et al. reported that RSV infection in pregnant women was associated with prematurity and low birth weight for gestational age (57). Again, 57% (4 out 7) of these infants tested positive for RSV infection during the 6 months of life. Subsequently, the same authors studied the serologic evidence of anti-RSV immunity in fetal cord blood of offspring born from women with RSV-mediated respiratory illness during the third trimester of pregnancy, and correlated the serology data with postnatal clinical outcomes (69). Anti-RSV IgG, IgA or IgM were detected in all cord blood serum samples drawn from babies born to RSV-infected mothers and experiencing adverse postnatal clinical outcomes. Fifty percent of seropositive newborns developed at least one respiratory tract sign/symptom, including respiratory distress syndrome (n=8), respiratory failure (n=3), and pneumonia (n=1). They also required more days on oxygen when compared to the control group (69).

## Severe Acute Respiratory Syndrome Coronavirus 2 (SARS-CoV-2) and Other Coronaviruses

The first official cases of coronavirus disease 2019 (COVID-19) caused by SARS-CoV-2 were described in Wuhan (Hubei, China) in December 2019, but the World Health Organization (WHO) declared the pandemic only several months later, on March 11, 2020. Since then, more than 260 million cases have been reported globally, with more than 5 million deaths (70). SARS-COV-2 is an enveloped RNA virus primarily transmitted *via* respiratory droplets, which, after infecting the nasal mucosal, spread to the lower airways binding to the angiotensin-converting enzyme 2 (ACE2) and the transmembrane serine protease 2 (TMPRSS2) (71). When ACE2 and TMPRSS2 are co-expressed, airway cells become more susceptible to virus entry (71). Whereas most infections are asymptomatic or mildly symptomatic, SARS-CoV-2 can trigger an explosive inflammatory response (cytokine storm) primarily mediated by IL-6, C-X-C motif chemokine 10 (CXCL10), and type 1 interferon, leading to acute respiratory distress syndrome (71).

Although initially pregnancy was not considered a risk factor for SARS-CoV-2 infection, more recent evidence suggests adverse clinical outcomes both in pregnant women and their offspring (72). It was reported that pregnant women show a clinical pattern similar to the general population, with 83% mild, 9.3% severe, and 4.7% critical disease, and severe pneumonia rate ranging from 0% to 33%. In the latter scenario, oxygen supplementation was needed in 5.2% to 100% of the cases, intubation in 1.7% of cases, and the rate of intensive care unit admission ranged from 6.9% to 21.1% (72–74). These data refer mostly to pregnant women in the third trimester of gestation; in contrast, only sparse evidence is available about the detection of COVID-19 in early pregnancy, and we have no data on the impact of SARS-CoV-2 infection during the first weeks of gestation (73–76). Accordingly, diagnosis of COVID-19 was made during the third trimester in 89.2% of cases, at delivery in 5.4% of cases, and after delivery in the remaining 5.4% of cases (76). Similar to the general population, the most common laboratory findings in pregnant women were high C-reactive protein (CRP) and procalcitonin levels (49-54%), lymphopenia (35%), and hyper-transaminasemia (16%) (74). Ground-glass appearance was observed on chest X-rays in 69% of cases, while other abnormalities on chest computed tomography were found in 65% of cases (77, 78).

The most common symptoms reported were fatigue, fever, cough, and anosmia with ageusia (74, 77, 78). Moreover, living in low- to middle-income countries and having a history of smoking, overweight or obesity, hypertension, diabetes, cardiovascular diseases, or chronic pulmonary diseases were risk factors for developing severe COVID-19 in pregnancy (74, 79–81). Co-morbidities increased the risk of intensive care admission and mortality significantly (80). In retrospective studies of pregnant women with confirmed or suspected COVID-19, the risk for pneumonia increased with maternal age above 35 years and lymphopenia, whereas it decreased with gestational age (76, 81). Surprisingly, high serum liver enzymes and D-dimers did not modify the risk of developing pneumonia (59, 74, 75). Overall, the prevalence of all-cause mortality was 0.63%, severe infection 13%, and admission to ICU 4% (74–76, 78).

SARS-CoV-2 infection in pregnancy, especially when complicated by pneumonia, was associated with higher rates of caesarean delivery and preeclampsia (70, 81, 82). The rate of caesarean delivery ranged from 42.9 to 100%, with the most common indications being maternal respiratory failure, fetal hypoxemia, and twin pregnancy (74, 78). In a systematic review and meta-analysis including 28 studies with 790,954 pregnant women, of which 15,524 diagnosed with SARS-CoV-2 infection, data showed a statistically significant increase of the risk of preeclampsia in pregnant women with COVID-19 when compared to controls without the infection. Moreover, severe clinical complications like eclampsia and HELLP (hemolysis, elevated liver enzymes and low platelets) syndrome were more frequent in pregnant women with symptomatic COVID-19 than those with asymptomatic disease (83).

A significant increase in stillbirth, ruptured ectopic pregnancies, maternal depression, and maternal deaths was also reported during the COVID-19 pandemic, but these findings came from studies with “critically low” overall quality due to lack or inconsistency in the selection of healthy controls, unreported or unclear definition of diseases, lack of clarity in reporting of outcomes, study design (e.g., retrospective analysis), and substantial statistical heterogeneity. Additionally, as an indirect consequence of the COVID-19 pandemic, changes in healthcare-seeking behavior and in the access to prenatal services have been reported as impacting negatively on the maternal outcomes (81).

Looking more specifically to the maternal-fetal interface, studies using precision-cut slices (PCSs) have shown that SARS-CoV-2 infection of the human placenta results in complete replication cycles with the release of infectious virus (84). Interestingly, the placenta’s susceptibility to SARS-CoV-2 replication was related to the expression of ACE2 and TMPRSS2 (84–91). A recent study also showed that ACE2-positive circulating immune cells of pregnant women with COVID-19 and chorioamnionitis could transfer the SARS-CoV-2 virus to the placenta, thus increasing the chance of vertical transmission (91). Several other investigators examined samples from mid-trimester placentae of women with COVID-19. However, whether SARS-CoV-2 isolation was due to primary infection or secondary to other placental pathology remains unclear. Importantly, when virus-like particles were detected in placental tissues, fetal tissues did not always show SARS-CoV-2 expression (84, 86–91). Other authors reported that exposure to SARS-CoV-2 causes neither cytotoxicity nor a proinflammatory cytokine response, and a significant expression in type-III IFN was also noted. Moreover, viral RNA and proteins were found in the syncytiotrophoblast, cytotrophoblasts, villous stroma, and possibly Hofbauer cells (84). SARS-CoV-2 functional modulation of macrophages localized on chorioamniotic membranes has also been reported, resulting in a mild inflammatory response systemically and at the maternal-fetal interface mediated by IL-8, IL-10, and IL-15 (92). Accordingly, histopathologic findings of abnormal maternal or fetal vascular perfusion were detected in 46% and 35% of analyzed placentas, respectively (86–91). Thus, the virus can cause vascular injury leading to ischemic damage of the placenta, affecting the fetus even without a direct infection (86–91).

On the other hand, the risk of vertical transmission from mother to fetus has consistently been reported as being relatively low (93). While the transfer of non-SARS-CoV-2-specific antibodies is conserved in COVID-19 positive mothers, the transfer of SARS-CoV-2-specific antibodies is significantly compromised during the third trimester of pregnancy compared to the second trimester (94). Because significant changes are occurring in antibody glycosylation, this could improve the ability of the innate immune system to contain the infection (94). Furthermore, Bordt et al. compared antibody and antiviral IFN responses in SARS-CoV-2-infected pregnant women versus uninfected controls and evaluated whether the fetal sex could affect the immune response. By quantifying anti-SARS-CoV-2 antibody titers and functions in the maternal and cord blood sera, the authors found that the mothers carrying male fetuses had lower maternal and cord blood titers than mothers carrying female pregnancies. Moreover, the transplacental transfer of IgG against other infectious agents, including influenza and pertussis, was not affected in male versus female pregnancies (95). No evidence of SARS-CoV-2-specific IgM in newborns was reported, supporting that vertical transmission of SARS-CoV-2 is uncommon (94–96).

A recent review warned that most of the studies reported only the proportion of positive newborns without assessing congenital, perinatal, or breast milk transmission, and even when these variables were included the sample size was generally small (93). Moreover, no consensus has been achieved regarding study protocols; for example, newborns enrolled in different studies were diagnosed with different COVID-19 tests. In most cases (75%), newborns were tested by reverse transcriptase-polymerase chain reaction (RT-PCR) of nasopharyngeal swabs, followed by RT-PCR of breast milk samples (60%), umbilical cord blood samples (53.3%), and amniotic fluid (46.7%). Also, most of the collected samples were discarded because they were contaminated or quantitatively insufficient (93, 97). Thus, no definitive evidence has been obtained so far about the true rate of COVID-19 vertical transmission (93, 98, 99).

Only one multicenter study conducted on 42 pregnant women with COVID-19 estimated the prevalence of neonatal infection was at 7.1%, although intrapartum or postpartum infections could not be excluded with certainty (100). Indeed, during labor infected women release aerosols and droplets that can potentially infect the newborn immediately after the birth. Moreover, stools released during labor can reach the vaginal canal and infect the newborn’s oropharynx during vaginal birth (101). Conversely, the probability of postnatal SARS-CoV-2 infection acquired through lactation appears small because no replication-competent virus has been found in breast milk (101–103). Postnatal transmission of SARS-CoV-2 could also occur immediately after the birth when the newborn is exposed to the infected mother or caregivers (101–103). However, virologic and serologic tests might not be able to pinpoint the route of infection. As an example, RT-PCR may amplify contaminating viral fragments picked up during vaginal delivery or during postnatal care of the newborn. It has been suggested that at least 2 positive SARS-CoV-2 RT-PCR tests are needed to achieve diagnostic certainty, but 2 samples are not always easy to obtain. Regarding serologic testing, both false negative and false positive results are frequently reported; thus, when a first serological test is positive, a confirmatory test, e.g., a second serological test or a molecular diagnostic test, is recommended (104).

Although stillbirth, prematurity (ranging from 18.9% to 66%), asphyxia, respiratory distress (4.9%), large for gestational age, low birth weight, small for gestational age, multiple organ dysfunction syndrome, disseminated intravascular coagulation, and congenital abnormalities (3.3%) have been reported in babies delivered from COVID-19-infected mothers, experts’ opinions on this issue are not unanimous (97, 105–110). Also, the rate of NICU hospitalization varied significantly, ranging from 0% to 8% of newborns from mothers with COVID-19 (97, 105–110), and it should be emphasized that admission to the NICU did not occur necessarily because of clinical indication, but also to provide isolation from other newborns and maternal contact despite the patient appeared asymptomatic and clinically stable (105–110). The prevalence of neonatal death in the offspring of women diagnosed with COVID-19 during pregnancy has been estimated ranging from 0% to 7%. However, the relationship between infection neonatal mortality remains unclear because all deaths occurred in preterm newborns (110–112). It has been hypothesized that immune changes occurring in babies born from COVID-19 positive mothers can influence the perinatal outcomes. In this context, Gee et al. (96) reported high levels of plasma cytokines, NK cells, and Treg cells in babies born from mothers with recent or acute infection, compared to those born to recovered or uninfected mothers. Conversely, similar numbers of B cells, CD4+ T cells, and CD8+ T cells levels were detected among the two groups (96). At any rate, neonatal mortality seems higher in pregnant women with COVID-19 pneumonia, possibly because the critical respiratory status of the mother compromises placental blood flow (110–112). Overall, analysis of the current literature suggests that the risk of vertical transmission of SARS-CoV-2 is very low and has been confirmed definitively only in a small minority of cases (110–112).

## Other Coronaviruses

Before the COVID-19 pandemic, the severity and consequences of coronavirus-induced infections, such as severe acute respiratory syndrome (SARS)-CoV and the Middle East respiratory syndrome (MERS)-CoV, had been widely investigated in pregnant women. Both coronaviruses caused severe maternal morbidity and mortality, significant obstetrical complications, and increased neonatal morbidity and mortality (113). Probably, impaired antigen presentation, poor T cell responses, and declined anti-viral immunity were all contributing to adverse maternal and neonatal outcomes (114). Also, during physiologic pregnancy, trophoblast cells at the maternal–fetal interface express Toll-like receptors (TLRs), which, after interaction with bacterial or viral antigens, induce the release of type-I IFN (115). When a viral infection occurs, type-I IFN exerts proinflammatory effects resulting in pregnancy complications (3). Finally, the placenta of women infected by SARS during the third trimester of pregnancy showed signs of thrombotic vasculopathy, which was consistent with reduced fetal perfusion (116).

## Conclusions

Profound physiological changes occur in pregnancy, involving the immune, respiratory, cardiovascular, coagulation, and hormonal systems. The combination of these events makes pregnant women more susceptible to respiratory pathogens, such as influenza, RSV, and SARS-CoV-2 (1, 2). Depending on the viral agent, the timing of infection during gestation, and the efficiency of viral clearance by the host immune system, the infection can follow different trajectories and the severity of infection in the mother and offspring can range from mild to severe, and even fatal (1, 2). Thus, in addition to the seasonal epidemics caused by influenza and RSV, now also SARS-CoV-2 is impacting public and global health dramatically. Furthermore, despite the time passed since the first reports and great efforts made by scientists worldwide, the mechanisms of vertical transmission and the acute and long-term consequences of perinatal influenza, RSV, and COVID-19 remain unclear.

Therefore, it is extremely important to expand basic, translational and clinical research efforts in this field. In the meantime, we have the opportunity to act on the interaction between viral pathogens, pregnant women, and their offspring by already available tools for passive and active viral prophylaxis. Vaccines and monoclonal antibodies administered before and during pregnancy provide effective protection against many common viral agents, not only for the mother but also for the fetus, and may have long-term benefits for the newborn that last into childhood and even adulthood. Indeed, active transplacental transport during the third trimester of pregnancy allows maternal antibodies to cross the placenta, reach the fetus, and provide protection during the first months of life when respiratory pathogens cause the most severe infections. Moreover, maternal antibodies can be transferred to the newborn after delivery *via* the breast milk, thereby extending the duration of immune protection during a critical window when the baby cannot mount a fully protective immune response against many pathogens, including viral agents.

## Author Contributions

All authors made substantial contribution to the conception of the work. SM and GP reviewed the literature on the subject. SM and GP drafted the final version of the manuscript. SL, FR, TH,and MP revised it critically for important intellectual content. All authors finally approved the version to be published and agreed to be accountable for all aspects of the work in ensuring that questions related to the accuracy or integrity of any part of the work are appropriately investigated and resolved.

## Conflict of Interest

The authors declare that the research was conducted in the absence of any commercial or financial relationships that could be construed as a potential conflict of interest.

## Publisher’s Note

All claims expressed in this article are solely those of the authors and do not necessarily represent those of their affiliated organizations, or those of the publisher, the editors and the reviewers. Any product that may be evaluated in this article, or claim that may be made by its manufacturer, is not guaranteed or endorsed by the publisher.
